# Supplementary material for: Comparing Efficacy of Erlotinib and Bevacizumab Combination with Erlotinib Monotherapy in Patients with Advanced Non-Small Cell Lung Cancer (NSCLC): A Systematic Review and Meta-Analysis
Source: Diseases. 2023 Oct 23;11(4):146. doi: 10.3390/diseases11040146 (PMC10594499; doi:10.3390/diseases11040146)
Supplement: Supplementary file 1 [file diseases-11-00146-s001.zip › Supplimentary Figure S1.pdf]

S1A. risk ratio of grade ≥ 3 AEs, random effect model [30,32–34,36–38]

Study or Subgroup	ERL+BEV Events	ERL+BEV Total	ERL Events	ERL Total	Weight	Risk Ratio M-H, Random, 95% CI
Herbst et al., 2011 [30]	208	313	165	313	17.3%	1.26 [1.11, 1.44]
Lee et al., 2023 [38]	29	64	6	63	9.1%	4.76 [2.12, 10.67]
Piccirillo et al., 2022 [33]	45	80	39	79	15.7%	1.14 [0.85, 1.53]
Saito et al., 2019 [32]	98	112	53	114	16.6%	1.88 [1.53, 2.32]
Seto et al., 2014 [36]	71	75	24	77	15.2%	3.04 [2.17, 4.25]
Stinchcombe et al., 2019 [34]	26	43	15	45	13.3%	1.81 [1.12, 2.93]
Zhou et al., 2021 [37]	63	157	15	153	12.8%	4.09 [2.44, 6.87]
Total (95% CI)		844		844	100.0%	2.09 [1.47, 2.97]
Total events	540		317			

Heterogeneity: $Tau^2 = 0.18$; $Chi^2 = 57.22$, df = 6 ($P < 0.00001$); $I^2 = 90\%$
Test for overall effect: Z = 4.11 ($P < 0.0001$)

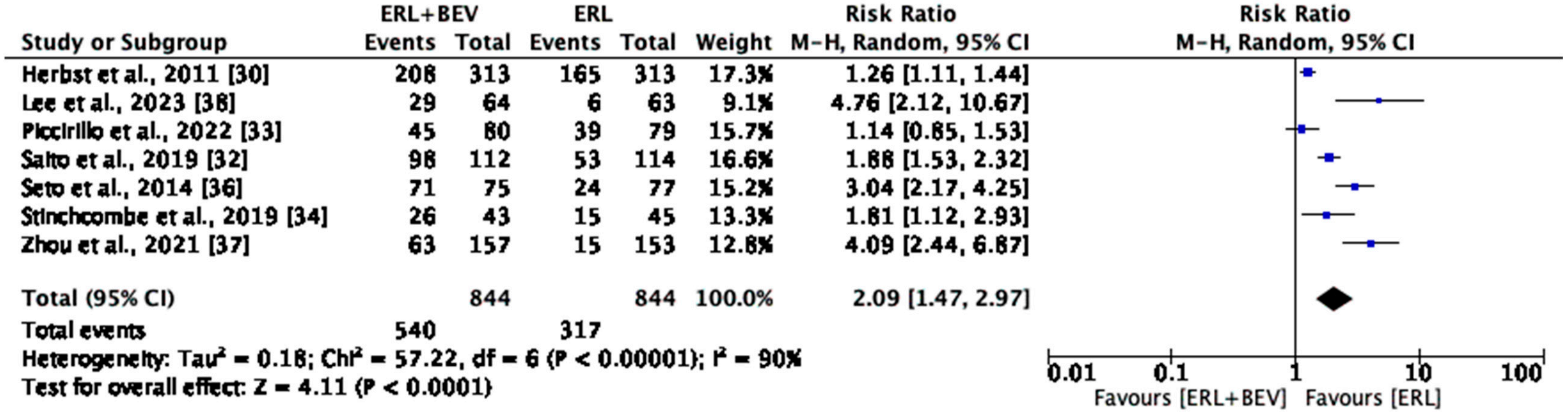

S1B. risk ratio for skin rash, random effect model [30,32–34,36–38]

Study or Subgroup	ERL+BEV Events	ERL+BEV Total	ERL Events	ERL Total	Weight	Risk Ratio M-H, Random, 95% CI
Herbst et al., 2011 [30]	49	313	19	313	16.5%	2.58 [1.55, 4.28]
Lee et al., 2023 [38]	11	64	3	63	9.1%	3.61 [1.06, 12.33]
Piccirillo et al., 2022 [33]	73	80	70	79	19.7%	1.03 [0.93, 1.14]
Saito et al., 2019 [32]	23	112	24	114	16.5%	0.98 [0.59, 1.62]
Seto et al., 2014 [36]	19	75	15	77	15.5%	1.30 [0.72, 2.36]
Stinchcombe et al., 2019 [34]	11	43	7	45	12.6%	1.64 [0.70, 3.85]
Zhou et al., 2021 [37]	8	157	5	153	10.2%	1.56 [0.52, 4.66]
Total (95% CI)		844		844	100.0%	1.53 [0.92, 2.52]
Total events	194		143			

Heterogeneity: $Tau^2 = 0.33$; $Chi^2 = 37.50$, df = 6 ($P < 0.00001$); $I^2 = 84\%$
Test for overall effect: Z = 1.65 ($P = 0.10$)

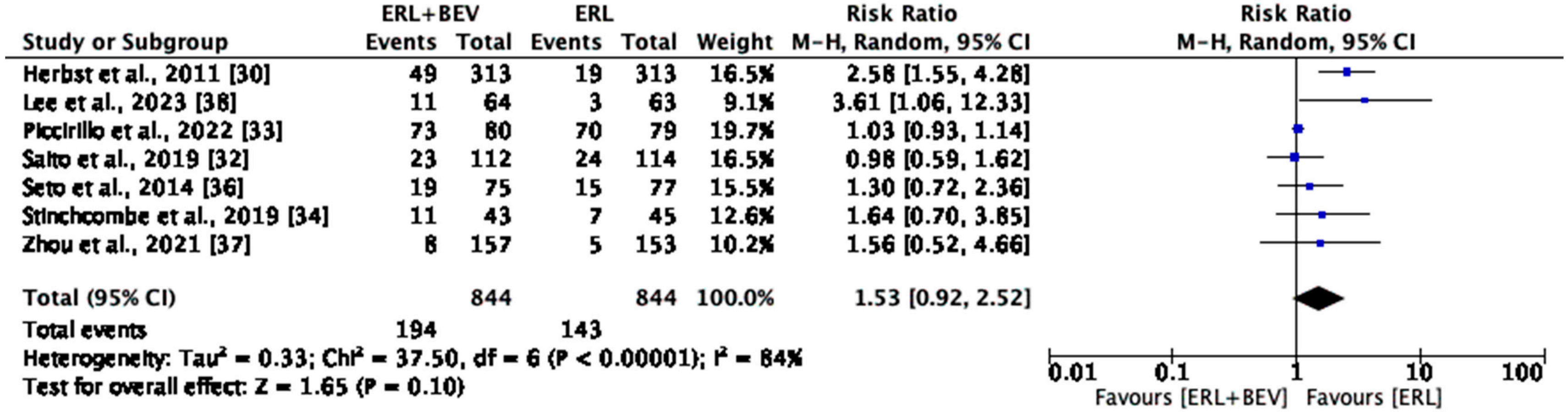

S1C. risk ratio for hypertension, random effect model [30,32–34,36–38]

Study or Subgroup	ERL+BEV Events	ERL+BEV Total	ERL Events	ERL Total	Weight	Risk Ratio M-H, Fixed, 95% CI
Herbst et al., 2011 [30]	15	313	4	313	12.8%	3.75 [1.26, 11.17]
Lee et al., 2023 [38]	9	64	0	63	1.6%	18.71 [1.11, 314.73]
Piccirillo et al., 2022 [33]	19	80	4	79	12.9%	4.69 [1.67, 13.17]
Saito et al., 2019 [32]	26	112	1	114	3.2%	26.46 [3.65, 191.71]
Seto et al., 2014 [36]	45	75	8	77	25.2%	5.78 [2.92, 11.42]
Stinchcombe et al., 2019 [34]	17	43	9	45	28.1%	1.98 [0.99, 3.95]
Zhou et al., 2021 [37]	29	157	5	153	16.2%	5.65 [2.25, 14.22]
Total (95% CI)		844		844	100.0%	5.15 [3.59, 7.39]
Total events	160		31			

Heterogeneity: $Chi^2 = 11.30$, $df = 6$ ($P = 0.08$); $I^2 = 47\%$
Test for overall effect: $Z = 8.91$ ($P < 0.00001$)

Risk Ratio
M-H, Fixed, 95% CI

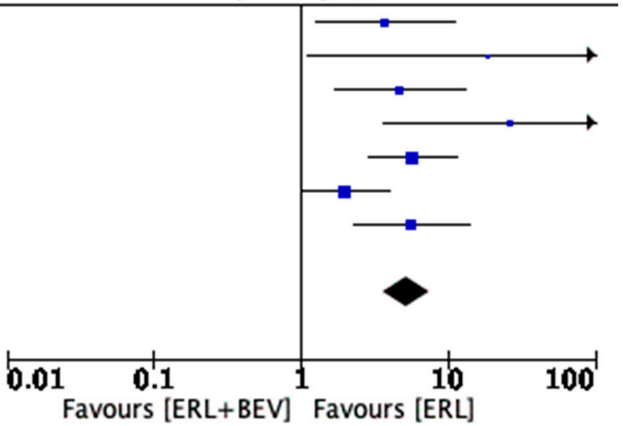

S1D. risk ratio for proteinuria, fixed effect model [32–34,36–38]

Study or Subgroup	ERL+BEV Events	ERL+BEV Total	ERL Events	ERL Total	Weight	Risk Ratio M-H, Fixed, 95% CI
Lee et al., 2023 [38]	5	64	0	63	12.6%	10.83 [0.61, 191.87]
Piccirillo et al., 2022 [33]	5	80	1	79	25.2%	4.94 [0.59, 41.32]
Saito et al., 2019 [32]	8	112	1	114	24.8%	8.14 [1.04, 64.04]
Seto et al., 2014 [36]	6	75	0	77	12.4%	13.34 [0.76, 232.75]
Stinchcombe et al., 2019 [34]	5	43	0	45	12.3%	11.50 [0.66, 201.90]
Zhou et al., 2021 [37]	11	157	0	153	12.7%	22.42 [1.33, 377.12]
Total (95% CI)		531		531	100.0%	10.54 [3.80, 29.20]
Total events	40		2			

Heterogeneity: $Chi^2 = 0.85$, $df = 5$ ($P = 0.97$); $I^2 = 0\%$
Test for overall effect: $Z = 4.53$ ($P < 0.00001$)

Risk Ratio
M-H, Fixed, 95% CI

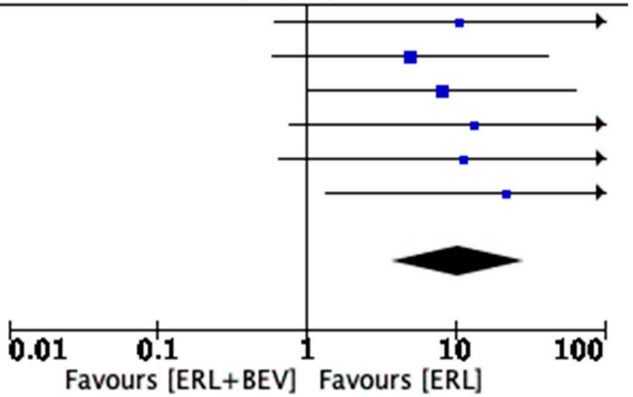

S1E. risk ratio for diarrhea, fixed effect model [32–34,36–38]

Study or Subgroup	ERL+BEV Events	ERL+BEV Total	ERL Events	ERL Total	Weight	Risk Ratio M-H, Fixed, 95% CI
Lee et al., 2023 [38]	4	64	3	63	19.7%	1.31 [0.31, 5.63]
Piccirillo et al., 2022 [33]	4	80	3	79	19.6%	1.32 [0.30, 5.69]
Saito et al., 2019 [32]	6	112	2	114	12.9%	3.05 [0.63, 14.81]
Seto et al., 2014 [36]	1	75	1	77	6.4%	1.03 [0.07, 16.12]
Stinchcombe et al., 2019 [34]	4	43	6	45	38.1%	0.70 [0.21, 2.30]
Zhou et al., 2021 [37]	4	157	0	153	3.3%	8.77 [0.48, 161.56]
Total (95% CI)		531		531	100.0%	1.53 [0.82, 2.86]
Total events	23		15			

Heterogeneity: $Chi^2 = 3.94$, df = 5 (P = 0.56); $I^2 = 0\%$
Test for overall effect: Z = 1.33 (P = 0.18)

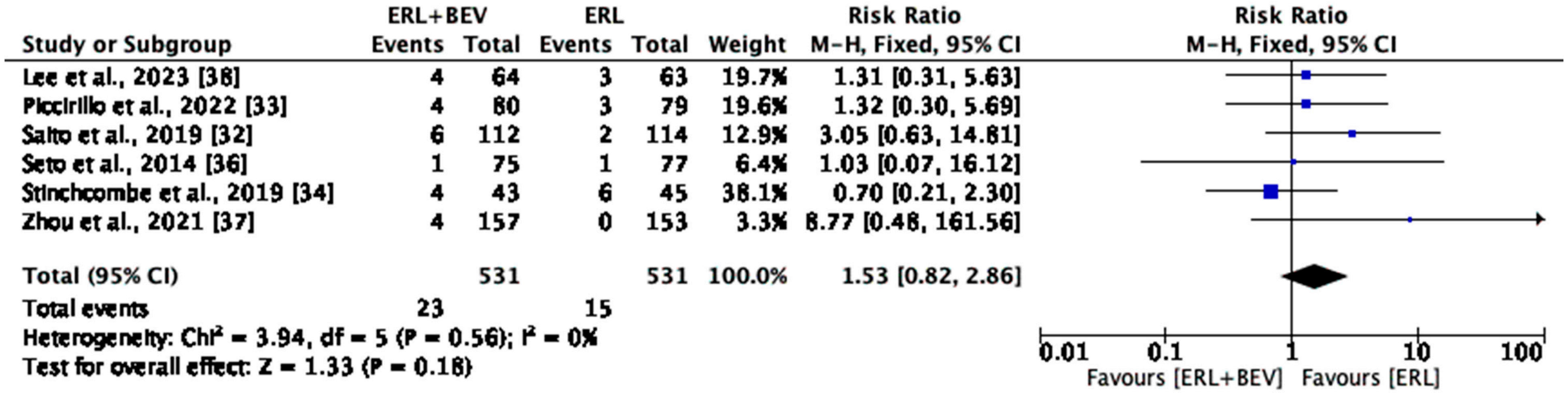

Supplementary Figure S1: meta-analyses of AEs: **(A)** risk ratio of grade ≥ 3 AEs, random-effect model; **(B)** risk ratio for skin rash, random-effect model; **(C)** risk ratio for hypertension, random-effect model; **(D)** risk ratio for proteinuria, fixed-effect model; **(E)** risk ratio for diarrhea, fixed-effect model; in patients with NSCLC receiving erlotinib-plus-bevacizumab combination and erlotinib monotherapy.
